# Associations between continuous glucose monitoring-derived metrics and arterial stiffness in Japanese patients with type 2 diabetes

**DOI:** 10.1186/s12933-020-01194-2

**Published:** 2021-01-07

**Authors:** Satomi Wakasugi, Tomoya Mita, Naoto Katakami, Yosuke Okada, Hidenori Yoshii, Takeshi Osonoi, Nobuichi Kuribayashi, Yoshinobu Taneda, Yuichi Kojima, Masahiko Gosho, Iichiro Shimomura, Hirotaka Watada

**Affiliations:** 1 Department of Metabolism & Endocrinology, Juntendo University Graduate School of Medicine, Hongo 2-1-1 Bunkyo-ku, Tokyo, Japan; 2 Department of Metabolic Medicine, Osaka University Graduate School of Medicine, 2-2 Yamadaoka, Suita, Osaka Japan; 3 Department of Metabolism and Atherosclerosis, Osaka University Graduate School of Medicine, 2-2 Yamadaoka, Suita, Osaka 565-0871 Japan; 4 First Department of Internal Medicine, School of Medicine, University of Occupational and Environmental Health, 1-1 Iseigaoka, Yahatanishi-ku, Kitakyushu, 807-8555 Japan; 5 Department of Medicine, Diabetology & Endocrinology, Juntendo Tokyo Koto Geriatric Medical Center, Shinsuna 3-3-20, Koto-ku, Tokyo, 136-0075 Japan; 6 Nakakinen Clinic, 745-5, Nakadai, Naka, Ibaraki 311-0113 Japan; 7 Misaki Naika Clinic, 6-44-9 Futawahigashi, Funabashi, Chiba 274-0805 Japan; 8 Taneda Clinic, Iwaki, Fukushima 973-8402 Japan; 9 Musashino Family Clinic, Minami 3-14-1, Yoshikawa, Saitama 342-0038 Japan; 10 Department of Biostatistics, Faculty of Medicine, University of Tsukuba, 1-1-1 Tennodai, Tsukuba, Ibaraki 305-8575 Japan

**Keywords:** Glucose variability, Continuous glucose monitoring, Arterial stiffness, Type 2 diabetes

## Abstract

**Background:**

Previous studies have suggested that high mean glucose levels and glycemic abnormalities such as glucose fluctuation and hypoglycemia accelerate the progression of atherosclerosis in patients with type 2 diabetes. Although continuous glucose monitoring (CGM) that could evaluate such glycemic abnormalities has been rapidly adopted, the associations between CGM-derived metrics and arterial stiffness are not entirely clear.

**Methods:**

This exploratory cross-sectional study used baseline data from an ongoing prospective, multicenter, observational study with 5 years of follow-up. Study participants included 445 outpatients with type 2 diabetes and no history of apparent cardiovascular disease who underwent CGM and brachial-ankle pulse wave velocity (baPWV) measurement at baseline. Associations between CGM-derived metrics and baPWV were analyzed using multivariate regression models.

**Results:**

In a linear regression model, all CGM-derived metrics were significantly associated with baPWV, but HbA1c was not. Some CGM-derived metrics related to intra-day glucose variability, hyperglycemia, and hypoglycemia remained significantly associated with baPWV after adjusting for possible atherosclerotic risk factors, including HbA1c. Based on baPWV ≥ 1800 cm/s as indicative of high arterial stiffness, multivariate logistic regression found that some CGM-derived metrics related to intra-day glucose variability and hyperglycemia are significantly associated with high arterial stiffness even after adjusting for possible atherosclerotic risk factors, including HbA1c.

**Conclusions:**

Multiple CGM-derived metrics are significantly associated with baPWV and high arterial stiffness in patients with type 2 diabetes who have no history of apparent cardiovascular disease. These metrics might be useful for identifying patients at high risk of developing cardiovascular disease.

## Background

Type 2 diabetes is an independent risk factor for cardiovascular disease (CVD), which is a major cause of death among patients with type 2 diabetes. Thus, achieving optimal glycemic control in patients with type 2 diabetes is indispensable to preventing CVD.

HbA1c is the gold standard for assessing glycemic control. It reflects the mean glucose level over the last 2–3 months. Some studies have demonstrated strong associations between HbA1c levels and diabetic complications [1, 2]. However, some large clinical trials have failed to show that intensive glycemic control assessed based on HbA1c has beneficial effects on CVD onset in patients with type 2 diabetes [3–5]. This would be most likely due to the fact that HbA1c does not provide all of the information on glycemic abnormalities, such intra-day and inter-day glucose variability and hypoglycemia, both of which may play an important role in the development of CVD. Thus, evaluating various aspects of glycemic status may help identify patients with a high probability of developing CVD. In this regard, continuous glucose monitoring (CGM) has emerged as an optimal method to obtain a more complete profile of blood glucose status that includes intra-day and inter-day glucose variations and patterns of hyperglycemia and hypoglycemia.

Importantly, recent clinical studies have suggested that glucose variability is more significantly associated with a higher subsequent incidence of myocardial infarction, acute heart failure, or cardiac death than the degree of hyperglycemic exposure indicated by HbA1c levels in patients with both type 2 diabetes or acute myocardial infarction [6, 7]. However, these studies included patients hospitalized for the treatment of acute myocardial infarction. Thus, CGM-derived metrics of the study participants could have been substantially affected by their disease status, treatment, and hospital meals, among other factors. Accordingly, these data may not be generalizable to outpatients with type 2 diabetes during their usual living conditions. Accordingly, the association between CGM-derived metrics measured in outpatients with type 2 diabetes and future incidence of CVD has not been fully elucidated yet.

On the other hand, recent clinical studies have demonstrated that standard deviation (SD), mean amplitude of glycemic excursion (MAGE), and time in range (TIR), which reflect intra-day glucose variation, are significantly associated with carotid artery intima-media thickening [8, 9]. Other studies have demonstrated that MAGE is associated with the presence and severity of coronary artery disease and vascular endothelial function, respectively [10, 11]. These studies indicate an association between CGM-derived metrics and various aspects of atherosclerosis. Increased arterial wall stiffness reflects the state of atherosclerosis. However, the degree of arterial stiffness does not necessarily reflect arterial intima-media thickness or endothelial function. To assess arterial wall stiffness, brachial-ankle pulse wave velocity (baPWV) is a noninvasive parameter that is often used clinically. It is useful for evaluating the state of atherosclerosis and predicting CVD or mortality in patients with type 2 diabetes [12, 13]. However, the relationship between CGM-derived metrics and arterial stiffness has not yet been fully clarified.

In this explanatory study, we investigated the relationship between CGM-derived metrics and arterial stiffness in 445 patients with type 2 diabetes who do not have a history of apparent CVD.

## Research design and methods

### Study design

This study is an exploratory sub-analysis of an ongoing, observational, prospective cohort study that aims to investigate the relationship between glucose variability evaluated with CGM and the incidence of composite cardiovascular events over a 5-year follow-up period, as described previously [14]. This study is a cross-sectional study using baseline data from the cohort study. This study has been registered in the University Hospital Medical Information Network Clinical Trials Registry (UMIN-CTR), which is a non-profit organization in Japan that meets the requirements of the International Committee of Medical Journal Editors (UMIN000032325).

### Study population

The study population consists of Japanese patients with type 2 diabetes who regularly attend the outpatient diabetes clinics of 34 institutions listed in Additional file 1: Table S1. The study design, inclusion criteria, and exclusion criteria were published previously [14]. Briefly, outpatients aged ≥ 30 years and ≤ 80 years with stable diabetes control were included. Patients with a history of myocardial infarction, angina pectoris, cerebral stroke, cerebral infarction, or arteriosclerosis obliterans were excluded. A history of CVD was confirmed based on medical history and patient interview. Consecutive subjects were screened. Patients who meet the eligibility criteria were asked to participate in the present study. A total of 1000 outpatients with type 2 diabetes under stable control and with no history of apparent CVD was recruited between May 2018 and March 2019. One patient withdrew consent. Among the remaining 999 subjects, 446 who underwent baPWV assessment were included in this exploratory sub-analysis.

The protocol was approved by the institutional review board of each participating institution in compliance with the Declaration of Helsinki and current legal regulations in Japan. Written informed consent was obtained from all participants after a full explanation of the study.

### Biochemical tests

Blood samples were obtained at visits after overnight fasting. Renal function, lipid levels, and HbA1c (National Glycohemoglobin Standardization Program) were measured with standard techniques. Urinary albumin excretion (UAE) was measured using a latex agglutination assay on a spot urine sample. Estimated glomerular filtration rate (eGFR) was calculated using a formula [15].

### CGM with the FreeStyle Libre Pro Device

The FreeStyle Libre Pro CGM (FLP-CGM) device (Abbott Japan, Tokyo, Japan), which measures glucose levels every 15 min for up to 14 days, was used in this study as previously reported [14]. Other than FLP-CGM use, there were no restrictions on participants’ daily lives. Downloaded data sets were further analyzed. Glucose variability was assessed based on MAGE [16], SD, and coefficient of variation (CV). MAGE was calculated as the arithmetic mean of the differences between consecutive peaks and nadirs, provided that the differences are greater than one SD of the mean glucose value. CV (%) was calculated by dividing SD by the mean of the corresponding glucose readings. The original statistical analysis plan (SAP) for this study was reported in the initial study protocol [14]. After the SAP was published, the Advanced Technologies & Treatments for Diabetes Congress proposed some CGM-derived metrics as useful clinical targets that complement HbA1c [17]. Thus, we updated the SAP by adding some CGM-derived metrics to this study prior to database lock. Mean glucose was calculated from data collected during FLP-CGM use. TIR was defined as the percentage of time spent in the target range between 3.9 and 10.0 mmol/L (time in range, TIR 3.9–10 mmol/L), time above target glucose range (TAR > 10 mmol/L, TAR > 13.9 mmol/L), and time below target glucose range (TBR < 3.9 mmol/L, TBR < 3.0 mmol/L). Low blood glucose index (LBGI) and high blood glucose index (HBGI) formulae were implemented by converting glucose values into risk scores [18]. In addition, mean of daily differences (MODD) [19] in glucose levels and interquartile range (IQR) were calculated to assess inter-day glucose variability. MODD was calculated as the mean of the absolute difference between glucose levels measured at the same time on 2 consecutive days. IQR was calculated using values from the same time of day during the monitoring period. Since a previous study demonstrated that FLP-CGM was less accurate during the first 24 h (from the first day to the second day) after insertion and during the last four days of its 14-day lifetime [20], we analyzed FLP-CGM data over the middle 8-day period.

### Measurement of baPWV

At baseline, baPWV was measured using an automatic waveform analyzer (BP-203RPE form; Colin Medical Technology, Komaki, Japan), as described previously [21]. Briefly, measurement was performed in the supine position after 5 min of bed rest. Cuffs for occlusion and monitoring were placed snugly around both arms and both ankles. The pressure waveforms were then recorded simultaneously from the brachial arteries using the oscillometric method. All scans were conducted by well-trained observers at each institution. A previous study confirmed that baPWV measurements have high reproducibility [22]. Subjects with an ankle-brachial index ≤ 0.90 were considered to have peripheral artery disease; baPWV data of these individuals were excluded from this study. Based on these exclusion criteria, 1 of 446 patients who underwent baPWV assessment was excluded. BaPWV assessment was conducted at baseline with a ± 10-week buffer period, but it was not performed while patients were wearing FLP sensors.

### Statistical analysis

Results are presented as mean ± SD or medians (range) for continuous variables or numbers (proportion) of patients for categorical variables. Comparisons between two groups were analyzed with Student’s *t* test and Wilcoxon’s rank sum test for continuous data or the Chi square test or Fisher’s exact test for categorical data as appropriate.

Subjects were categorized into two groups based on the baPWV value of 1800 cm/s. We used this cutoff value recommended by the Japanese Circulation Society to identify subjects who are at high risk for developing CVD [23]. High arterial stiffness was defined as baPWV ≥ 1800 cm/s and low arterial stiffness was defined as baPWV < 1800 cm/s.

Multivariate linear regression analysis was performed to investigate whether FLP-CGM-derived metrics are associated with baPWV when it was treated as continuous variable. In addition, multivariate logistic regression analysis was performed to investigate whether FLP-CGM-derived metrics are associated with high baPWV when it was treated as a dichotomous variable. Potential conventional risk factors evaluated with clinical, biochemical, or metabolic testing were included in the models based on clinical judgment. All statistical tests were two-sided with a 5% significance level. All analyses were performed using SAS software version 9.4 (SAS Institute, Cary, NC).

## Results

### Baseline clinical characteristics

The baseline clinical characteristics of the 445 patients with type 2 diabetes are summarized in Table 1. Mean age was 65.9 ± 9.0 years, 67.6% were male, HbA1c was 7.08 ± 0.78% (53.9 ± 8.6 mmol/mol), and estimated duration of type 2 diabetes was 13.5 ± 8.3 years.

**Table 1:** Patient demographic and background characteristics (n = 445)

Parameter	
Age (years)	65.9 ± 9.0
Male gender (%)	301 (67.6)
Body mass index (kg/m^2^)	24.5 ± 3.8
Estimated duration of diabetes (years)	13.5 ± 8.3
Current smoker (%)	92 (20.7)
Systolic blood pressure (mmHg)	132.5 ± 14.8
Diastolic blood pressure (mmHg)	77.1 ± 11.2
HbA1c (%)	7.1 ± 0.8
HbA1c (mmol/mol)	53.9 ± 8.6
Total cholesterol (mmol/L)	4.92 ± 0.78
LDL cholesterol (mmol/L)	2.77 ± 0.64
HDL cholesterol (mmol/L)	1.55 ± 0.40
Triglycerides (mmol/L)	1.40 ± 1.0
Uric acid (μmol/L)	310.6 ± 73.5
Estimated glomerular filtration rate (mL/min/1.73 m^2^)	70.8 ± 18
FLP-CGM-derived metrics	
SD (mmol/L)	2.02 ± 0.66
CV (%)	25.6 ± 5.8
MAGE (mmol/L)	5.44 ± 2.09
TIR3.9–10 mmol/L (%)	78.7 ± 19.7
TAR > 10 mmol/L (%)	19.6 ± 20.1
TAR > 13.9 mmol/L (%)	4.03 ± 9.11
TBR < 3.9 mmol/L (%)	1.77 ± 4.28
TBR < 3.0 mmol/L (%)	0.30 ± 1.34
LBGI	1.42 ± 1.60
HBGI	5.63 ± 4.64
MODD (mmol/L)	1.72 ± 0.62
IQR (mmol/L)	2.12 ± 0.77
baPWV (cm/s)	1706 ± 367
baPWV (cm/s) in males (n = 301)	1698 ± 345
baPWV (cm/s) in females (n = 144)	1722 ± 412

Data are mean ± SD or number of patients (%)

baPWV: brachial-ankle pulse wave velocity; CV: coefficient of variation; FLP-CGM: FreeStyle Libre Pro continuous glucose monitoring; HBGI: high blood glucose index; HDL: high-density lipoprotein; IQR: interquartile range; LBGI: low blood glucose index; LDL: low-density lipoprotein; MAGE: mean amplitude of glycemic excursion; MODD: mean of daily differences; SD: standard deviation; TAR: time above range; TBR: time below range; TIR: time in range

### Relationship between FLP-CGM-derived metrics and baPWV

We investigated the relationship between FLP-CGM-derived metrics and baPWV in patients with type 2 diabetes when baPWV was treated as continuous variable. In a linear regression model, all calculated FLP-CGM-derived metrics were significantly associated with baPWV, although no significant association was observed between HbA1c and baPWV (Model 1 in Table 2). In Model 2, which adjusted for age and gender, all the aforementioned associations remained significant. After adjusting for variables in Model 2 plus body mass index (BMI) and duration of diabetes (Model 3), the associations between FLP-CGM-derived metrics and baPWV remained significant, except for LBGI. Even after adjusting for variables in Model 3 plus HbA1c, systolic blood pressure (BP), lipid parameters, uric acid, eGFR, UAE, smoking status, use of insulin therapy, use of angiotensin-converting enzyme (ACE) inhibitors and/or angiotensin II receptor blockers (ARBs), use of statins, use of anti-platelet agents, and presence of diabetic retinopathy (Model 4), the associations between the FLP-CGM-derived metrics of mean glucose, SD, CV, MAGE, TIR 3.9–10 mmol/L, TAR > 10 mmol/L, TAR > 13.9 mmol/L, TBR < 3.9 mmol/L, TBR < 3.0 mmol/L, and HBGI and baPWV remained significant (Table 2).

**Table 2:** Associations between FLP-CGM-derived metrics and branchial-ankle pulse wave velocity

Parameter	Regression coefficient (95% CI)	*P* value
Mean glucose (1 mmol/L increase)		
Model 1	25.0 (5.9 to 44.2)	0.011
Model 2	26.8 (9.9 to 43.6)	0.002
Model 3	26.7 (9.9 to 43.5)	0.002
Model 4	36.3 (10.3 to 62.5)	0.006
SD (mmol/L) (1 mmol/L increase)		
Model 1	137.2 (86.5 to 187.9)	< 0.001
Model 2	102.3 (56.8 to 147.9)	< 0.001
Model 3	93.0 (46.9 to 139.0)	< 0.001
Model 4	91.7 (36.5 to 146.8)	0.001
CV (%) (1% increase)		
Model 1	14.6 (8.9 to 20.3)	< 0.001
Model 2	8.6 (3.3 to 13.8)	0.001
Model 3	7.0 (1.6 to 12.4)	0.012
Model 4	5.9 (0.5 to 11.4)	0.034
MAGE (1 mmol/L increase)		
Model 1	39.7 (23.7 to 55.7)	< 0.001
Model 2	28.3 (13.9 to 42.7)	< 0.001
Model 3	26.3 (11.9 to 40.7)	< 0.001
Model 4	26.6 (10.4 to 42.8)	0.001
TIR3.9–10 mmol/L (10% increase)		
Model 1	− 33.5 (− 50.6 to − 16.5)	< 0.001
Model 2	− 29.8 (− 44.9 to − 14.7)	< 0.001
Model 3	− 28.8 (− 43.9 to − 13.7)	< 0.001
Model 4	− 37.1 (− 59.5 to − 14.8)	0.001
TAR > 10 mmol/L (1% increase)		
Model 1	2.51 (0.82 to 4.20)	0.004
Model 2	2.36 (0.86 to 3.85)	0.002
Model 3	2.31 (0.82 to 3.80)	0.002
Model 4	3.02 (0.72 to 5.33)	0.010
TAR > 13.9 mmol/L (1% increase)		
Model 1	4.92 (1.81 to 8.66)	0.010
Model 2	5.50 (2.22 to 8.79)	0.001
Model 3	5.61 (2.35 to 8.87)	< 0.001
Model 4	5.72 (1.41 to 10.0)	0.009
TBR < 3.9 mmol/L (1% increase)		
Model 1	16.1 (8.2 to 24.0)	< 0.001
Model 2	11.7 (4.6 to 18.7)	0.001
Model 3	10.3 (3.2 to 17.3)	0.004
Model 4	8.3 (1.19 to 15.5)	0.022
TBR < 3.0 mmol/L (1% increase)		
Model 1	52.1 (28.2 to 77.9)	< 0.001
Model 2	45.1 (23.0 to 67.1)	< 0.001
Model 3	41.5 (19.4 to 63.6)	< 0.001
Model 4	30.5 (8.3 to 52.7)	0.007
LBGI (1 unit increase)		
Model 1	25.6 (4.26 to 46.9)	0.019
Model 2	14.3 (− 4.8 to 33.3)	0.142
Model 3	9.1 (− 10.1 to 28.4)	0.350
Model 4	4.1 (− 16.5 to 24.6)	0.699
HBGI (1 unit increase)		
Model 1	12.6 (5.3 to 19.9)	< 0.001
Model 2	11.9 (5.5 to 18.3)	< 0.001
Model 3	11.6 (5.2 to 18.0)	< 0.001
Model 4	13.8 (4.7 to 23.0)	0.004
MODD (1 mmol/L increase)		
Model 1	64.5 (9.1 to 119.9)	0.023
Model 2	69.6 (20.7 to 118.3)	0.005
Model 3	58.4 (8.8 to 108.0)	0.021
Model 4	21.3 (− 42.0 to 84.7)	0.508
IQR (1 mmol/L increase)		
Model 1	45.7 (1.5 to 89.9)	0.043
Model 2	57.0 (18.1 to 95.8)	0.004
Model 3	48.7 (9.3 to 88.2)	0.015
Model 4	28.9 (− 22.1 to 80.0)	0.266
HbA1c (1% increase)		
Model 1	7.7 (− 36.2 to 51.4)	0.734
Model 2	22.7 (− 16.0 to 61.4)	0.250
Model 3	20.8 (− 18.2 to 59.8)	0.295
Model 4 (excluding HbA1c)	− 15.2 (− 55.7 to 25.2)	0.459

Model 1: crude

Model 2: adjusted for age and gender

Model 3: adjusted for variables in Model 2 plus BMI, and duration of diabetes

Model 4: adjusted for variables in Model 3 plus HbA1c, systolic blood pressure, total cholesterol, HDL cholesterol, logarithm of triglycerides, serum uric acid, estimated glomerular filtration rate, logarithm of urinary albumin excretion, presence of diabetic retinopathy, smoking status (never smoker, previous smoker, or current smoker), use of insulin therapy, use of angiotensin-converting enzyme inhibitors and/or angiotensin II receptor blockers, use of statins, and use of anti-platelet agents

CI: confidence interval; CV: coefficient of variation; FLP-CGM: FreeStyle Libre Pro continuous glucose monitoring; HBGI: high blood glucose index; IQR: interquartile range; LBGI: low blood glucose index; MAGE: mean amplitude of glycemic excursion; MODD: mean of daily differences; SD: standard deviation; TAR: time above range; TBR: time below range; TIR: time in range

### Relationship between FLP-CGM-derived metrics and high arterial stiffness

Next, 445 subjects were divided into a high arterial stiffness group (n = 149) and a low arterial stiffness group (n = 296) based on the cut-off baPWV value of 1800 cm/s. The clinical characteristics of the high and low arterial stiffness groups are summarized in Table 3. Subjects with high arterial stiffness were older, had diabetes for longer durations, lower BMI, higher systolic BP, higher UAE, higher prevalence of diabetic retinopathy, and lower eGFR. In addition, they were more frequently treated with sulfonylureas, dipeptidyl peptidase-4 inhibitors, insulin, and calcium channel blockers. There were significant differences in all FLP-CGM-derived metrics except for TBR < 3.9 mmol/L, TBR < 3.0 mmol/L, and LBGI between the two groups.

**Table 3:** Comparisons of clinical parameters between the higher and low arterial stiffness groups

Parameter	Low arterial stiffness (n = 296)	High arterial stiffness (n = 149)	*P* value
Age (years)	63.5 ± 9.2	70.7 ± 5.9	< 0.001
Male gender (%)	201 (67.9)	100 (67.1)	0.866
Body mass index (kg/m^2^)	24.8 ± 3.9	23.8 ± 3.4	0.007
Estimated duration of diabetes (years)	12.0 ± 7.4	16.4 ± 9.3	< 0.001
Diabetic retinopathy (%)	57 (19.3)	51 (34.2)	< 0.001
Smoking status (%) never/former/current smoker	123 (41.6)/102 (34.5)/71 (24)	65 (43.6)/63 (42.3)/21 (14)	0.040
Hypertension (%)	159 (53.7)	97 (65.1)	0.022
Systolic blood pressure (mmHg)	129.9 ± 12.4	137.6 ± 17.7	< 0.001
Diastolic blood pressure (mmHg)	77.3 ± 10.9	76.7 ± 11.8	0.560
HbA1c (%)	7.1 ± 0.83	7.1 ± 0.69	0.532
HbA1c (mmol/mol)	53.7 ± 9.0	54.2 ± 7.5	0.532
Total cholesterol (mmol/L)	4.93 ± 0.78	4.88 ± 0.78	0.523
LDL cholesterol (mmol/L)	2.78 ± 0.63	2.73 ± 0.64	0.492
HDL cholesterol (mmol/L)	1.55 ± 0.40	1.56 ± 0.40	0.757
Triglycerides (mmol/L)	1.39 ± 1.05	1.41 ± 1.04	0.459
Uric acid (μmol/L)	309.5 ± 74.5	312.7 ± 71.6	0.668
Estimated glomerular filtration rate (mL/min/1.73 m^2^)	72.9 ± 19	66.7 ± 17	< 0.001
Urinary albumin excretion (mg/g creatinine)	11.0 (0.9-1414.6)	21.6(1.9–7398.0)	< 0.001
Normoalbuminuria/microalbuminuria/macroalbuminuria (%)	230 (77.7)/51 (17.2)/15 (5.1)	92(61.7)/43(28.9)/14(9.4)	0.002
Use of oral glucose-lowering agents (%)	260 (88)	140 (94)	0.043
Metformin (%)	161 (54)	73 (49)	0.282
Sulfonylureas (%)	30 (10)	29 (20)	0.006
Glinides (%)	16 (5.4)	9 (6)	0.784
Dipeptidyl peptidase-4 inhibitors (%)	154 (52)	98 (65.8)	0.006
Sodium-glucose cotransporter 2 inhibitors (%)	73 (25)	29 (20)	0.218
Thiazolidinediones (%)	42 (14)	13 (9)	0.098
α-glucosidase inhibitors (%)	91 (31)	33 (22)	0.056
Glucagon-like peptide-1 antagonists (%)	12 (4)	8 (5.4)	0.628
Insulin (%)	31 (11)	32 (22)	0.002
Angiotensin-converting enzyme inhibitors (%)	8 (3)	10 (7)	0.071
Angiotensin II receptor blockers (%)	127 (43)	65 (44)	0.885
Calcium channel blockers (%)	64 (22)	53 (36)	0.002
Statins (%)	140 (47)	67 (45)	0.642
Antiplatelet agents (%)	10 (3)	9 (6)	0.217
FLP-CGM-derived metrics			
Mean glucose (mmol/L)	7.56 ± 1.68	8.13 ± 1.94	0.037
SD (mmol/L)	1.93 ± 0.60	2.22 ± 0.66	< 0.001
CV (%)	24.8 ± 5.62	27.4 ± 5.9	< 0.001
MAGE (mmol/L)	5.13 ± 1.88	6.06 ± 2.34	< 0.001
TIR3.9–10 mmol/L (%)	80.6 ± 19.5	74.8 ± 19.9	0.004
TAR > 10 mmol/L (%)	17.9 ± 19.7	22.9 ± 20.6	0.013
TAR > 13.9 mmol/L (%)	3.33 ± 7.98	5.41 ± 10.9	0.023
TBR < 3.9 mmol/L (%)	1.52 ± 3.52	2.27 ± 5.46	0.085
TBR < 3.0 mmol/L (%)	0.23 ± 1.23	0.42 ± 1.58	0.155
LBGI	1.34 ± 1.58	1.60 ± 1.63	0.103
HBGI	5.17 ± 4.22	6.56 ± 5.27	0.003
MODD (mmol/L)	1.67 ± 0.58	1.83 ± 0.68	0.011
IQR (mmol/L)	2.06 ± 0.71	2.24 ± 0.87	0.024

Data are mean ± SD, medians (range), or number of patients (%). Continuous data were compared using Student’s t-test or Wilcoxon’s rank sum test. Categorical data were compared using the Chi square test or Fisher’s exact test as appropriate

CV: coefficient of variation; FLP-CGM: FreeStyle Libre Pro continuous glucose monitoring; HBGI: high blood glucose index; HDL: high-density lipoprotein; IQR: interquartile range; LBGI: low blood glucose index; LDL: low-density lipoprotein; MAGE: mean amplitude of glycemic excursion; MODD: mean of daily differences; SD: standard deviation; TAR: time above range; TBR: time below range; TIR: time in range

Using this classification of baPWV, we investigated the relationship between FLP-CGM-derived metrics and high baPWV. In a multivariate logistic regression model, all FLP-CGM-derived metrics except for LBGI were significantly associated with high arterial stiffness, although no significant association was observed between HbA1c and arterial stiffness (Model 1 in Table 4). In Models 2 and 3, all FLP-CGM-derived metrics except for LBGI remained significantly associated with high baPWV. In Model 4, SD, CV, MAGE, TAR > 13.9 mmol/L, and HBGI remained significantly associated with high baPWV.

**Table 4:** Associations between FLP-CGM-derived metrics and high arterial stiffness

Parameter	Odds ratio (95% CI)	*P* value
Mean glucose (1 mmol/L increase)		
Model 1	1.12 (1.01–1.25)	0.039
Model 2	1.16 (1.03–1.32)	0.016
Model 3	1.17 (1.03–1.32)	0.014
Model 4	1.22 (0.98–1.52)	0.078
SD (mmol/L) (1 mmol/L increase)		
Model 1	1.99 (1.46–2.71)	< 0.001
Model 2	1.86 (1.33–2.59)	< 0.001
Model 3	1.73 (1.24–2.43)	0.001
Model 4	1.92 (1.22–3.03)	0.005
CV (%) (1% increase)		
Model 1	1.08 (1.04–1.12)	< 0.001
Model 2	1.06 (1.02–1.10)	0.003
Model 3	1.05 (1.01–1.09)	0.022
Model 4	1.05 (1.01–1.10)	0.024
MAGE (1 mmol/L increase)		
Model 1	1.24 (1.12–1.36)	< 0.001
Model 2	1.21 (1.08–1.34)	< 0.001
Model 3	1.19 (1.07–1.33)	0.001
Model 4	1.24 (1.08–1.42)	0.003
TIR 3.9–10 mmol/L (10% increase)		
Model 1	0.87 (0.79–0.96)	0.004
Model 2	0.86 (0.77–0.96)	0.007
Model 3	0.86 (0.77–0.96)	0.009
Model 4	0.85 (0.71–1.03)	0.096
TAR > 10 mmol/L (1% increase)		
Model 1	1.01 (1.00–1.02)	0.014
Model 2	1.01 (1.00–1.03)	0.013
Model 3	1.01 (1.00–1.03)	0.013
Model 4	1.02 (1.00–1.04)	0.097
TAR > 13.9 mmol/L (1% increase)		
Model 1	1.02 (1.00–1.05)	0.029
Model 2	1.03 (1.01–1.06)	0.005
Model 3	1.04 (1.01–1.06)	0.003
Model 4	1.05 (1.01–1.09)	0.016
TBR < 3.9 mmol/L (1% increase)		
Model 1	1.04 (0.99–1.09)	0.098
Model 2	1.02 (0.97–1.07)	0.453
Model 3	1.01 (0.96–1.06)	0.862
Model 4	1.00 (0.94–1.07)	0.914
TBR < 3.0 mmol/L (1% increase)		
Model 1	1.10 (0.96–1.28)	0.179
Model 2	1.07 (0.92–1.25)	0.367
Model 3	1.04 (0.90–1.21)	0.609
Model 4	1.00 (0.84–1.20)	0.968
LBGI (1 unit increase)		
Model 1	1.10 (0.98–1.24)	0.110
Model 2	1.06 (0.93–1.21)	0.384
Model 3	1.02 (0.89–1.16)	0.790
Model 4	1.00 (0.85–1.18)	0.973
HBGI (1 unit increase)		
Model 1	1.06 (1.02–1.11)	0.004
Model 2	1.08 (1.03–1.13)	0.002
Model 3	1.08 (1.03–1.13)	0.002
Model 4	1.10 (1.02–1.19)	0.013
MODD (1 mmol/L increase)		
Model 1	1.50 (1.09–2.05)	0.012
Model 2	1.71 (1.20–2.44)	0.003
Model 3	1.59 (1.11–2.29)	0.013
Model 4	1.40 (0.81–2.29)	0.239
IQR (1 mmol/L increase)		
Model 1	1.33 (1.03–1.71)	0.026
Model 2	1.53 (1.15–2.04)	0.003
Model 3	1.45 (1.08–1.94)	0.013
Model 4	1.40 (0.91–2.12)	0.129
HbA1c (1% increase)		
Model 1	1.08 (0.84–1.39)	0.531
Model 2	1.21 (0.91–1.62)	0.193
Model 3	1.20 (0.89–1.62)	0.228
Model 4 (excluding HbA1c)	0.93 (0.64–1.35)	0.707

Model 1: crude

Model 2: adjusted for age and gender

Model 3: adjusted for variables in Model 2 plus BMI, and duration of diabetes

Model 4: adjusted for variables in Model 3 plus HbA1c, systolic blood pressure, total cholesterol, HDL cholesterol, logarithm of triglycerides, serum uric acid, estimated glomerular filtration rate, logarithm of urinary albumin excretion, presence of diabetic retinopathy, smoking status (never smoker, previous smoker, or current smoker), use of insulin therapy, use of angiotensin-converting enzyme inhibitors and/or angiotensin II receptor blockers, use of statins, and use of anti-platelet agents

CI: confidence interval; CV: coefficient of variation; FLP-CGM: FreeStyle Libre Pro continuous glucose monitoring; HBGI: high blood glucose index; IQR: interquartile range; LBGI: low blood glucose index; MAGE: mean amplitude of glycemic excursion; MODD: mean of daily differences; SD: standard deviation; TAR: time above range; TBR: time below range; TIR: time in range

## Discussion

Recent studies have demonstrated that large glucose fluctuations are associated with a higher subsequent incidence of myocardial infarction, acute heart failure, or cardiac death in patients with acute myocardial infarction [7] and patients with both type 2 diabetes and acute myocardial infarction [6], respectively. However, it is still largely unknown whether CGM-derived metrics are associated with a higher subsequent incidence of CVD or atherosclerosis, including arterial stiffness in patients with type 2 diabetes and no history of apparent CVD. In this study, we demonstrated that most FLP-CGM-derived metrics are significantly associated with arterial stiffness, even after adjusting for various risk factors including HbA1c levels in 445 outpatients with type 2 diabetes and no history of apparent CVD. Notably, some FLP-CGM-derived metrics related to inter-day glucose variability and hyperglycemia identified subjects with high arterial stiffness who were at high risk for developing CVD.

In patients with type 2 diabetes, the main cause of arterial stiffness is damage to vascular walls caused by prolonged hyperglycemia. In fact, previous cross-sectional studies have indicated that higher HbA1c levels are associated with increased arterial stiffness in patients with type 2 diabetes [24, 25]. In contrast, HbA1c was not associated with arterial stiffness in this study, a finding that was consistent with a different previous study [26]. On the other hand, this study found significant associations between FLP-CGM-derived metrics related to hyperglycemia such as TAR > 13.9 mmol/L and HBGI. Unlike HbA1c, these parameters reflect remarkable hyperglycemia and were not affected by hypoglycemia. Thus, our data do not contradict the fact that the main cause of arterial stiffness is damage to vascular walls caused by prolonged hyperglycemia in patients with type 2 diabetes.

Previous cross-sectional studies have demonstrated that CGM-derived metrics related to intra-day glucose variability are associated with carotid atherosclerosis, coronary atherosclerosis, or endothelial dysfunction in patients with type 2 diabetes [8–11]. This study also found that SD, CV, and MAGE are significantly associated with arterial stiffness; these variables reflect another aspect of atherosclerosis in patients with type 2 diabetes. Various factors are involved in the progression of arterial stiffness in patients with type 2 diabetes. Indeed, previous cross-sectional studies have demonstrated that conventional atherosclerotic risk factors such as age, BMI, duration of type 2 diabetes, glycemic control, dyslipidemia, systolic BP, eGFR, elevated uric acid levels, and albuminuria [27–29] are associated with arterial stiffness in patients with type 2 diabetes. Intriguingly, in this study FLP-CGM-derived metrics related to intra-day glucose variability were significantly associated with degree of arterial stiffness, even after adjusting for those possible conventional atherosclerotic risk factors. Taken together, this study highlighted the importance of intra-day glucose variability in terms of assessing the risk of arterial stiffness.

A recent study demonstrated that the incremental glucose peak, an indicator of glucose variability, during an oral glucose tolerance test is associated with arterial stiffness in patients with type 2 diabetes [30]. Another study showed that impaired glucose regulation characterized by fasting or post-challenge hyperglycemia is associated with arterial stiffness [31]. However, whether oral glucose tolerance test-derived incremental glucose peak reflects the pattern of glucose variability during usual living conditions has not yet been clarified. In this regard, this is the first study to provide evidence that FLP-CGM-derived metrics related to intra-day glucose variability evaluated during usual living conditions are significantly associated with arterial stiffness. Although the exact mechanism of how glucose variability contributes to arterial stiffness remains unclear, we propose the following possible scenarios. Previous studies have shown that glucose variability induces inflammation and overproduction of oxidative stress to a greater extent than chronic persistent hyperglycemia [32, 33], leading to advanced AGE formation. The formation of AGEs is considered to be involved in arterial stiffness through cross-linking of collagen molecules and a subsequent loss of collagen elasticity [34]. Accordingly, vascular walls may be damaged by glucose variability more than by chronic persistent hyperglycemia.

In this study, FLP-CGM-derived metrics related to hypoglycemia were associated with arterial stiffness. Similarly, some recent studies have demonstrated that the acute effects of hypoglycemia include inflammation and endothelial injury in patients with type 1 diabetes [35, 36]. In addition, a cross-sectional study demonstrated that repeated episodes of hypoglycemia are associated with preclinical atherosclerosis as evaluated by carotid and femoral ultrasonography and measurement of flow-mediated brachial dilatation in patients with type 1 diabetes [37]. Furthermore, we previously reported that a higher frequency of hypoglycemic episodes is associated with progression of carotid atherosclerosis in patients with type 2 diabetes [38]. Accordingly, physicians need to help prevent hypoglycemic episodes through assessing the risks of hypoglycemia with CGM in order to minimize arterial stiffness, especially in patients who have difficulty detecting or who are completely unaware of hypoglycemia.

As discussed above, this study demonstrated that FLP-CGM-derived metrics are significantly associated with arterial stiffness. On the other hand, it is more important to screen for patients with a high potential of developing CVD in order to reduce the incidence of CVD. To achieve this goal, the Japanese Circulation Society proposed baPWV of 1800 cm/s as the cutoff value to identify subjects who are at high risk for developing CVD based on the results of several studies [39]. Based on this cutoff value, approximately 33% of study participants were defined as being at high risk for CVD. Subjects with high arterial stiffness were older, had longer duration of diabetes, lower BMI, higher systolic BP, higher UAE, higher prevalence of diabetic retinopathy, and lower eGFR. They were more frequently treated with insulin and calcium channel blockers. Even after adjusting for these confounding factors, FLP-CGM-derived metrics related to intra-day glucose variability such as SD, CV, MAGE, and metrics related to hyperglycemia such as TAR > 13.9 mmol/L and HBGI were significantly associated with high arterial stiffness. Given that FLP-CGM-derived metrics related to hypoglycemia, such as TIR 3.9–10 mmol/L and TAR > 10 mmol/L were not associated with high arterial stiffness, high postprandial glucose excursion amplitude may be a major contributor to increased arterial stiffness. Thus, based on FLP-CGM-derived metrics, focusing on reducing the amplitude of postprandial glucose excursion may be important to reducing the risk of both arterial stiffness and CVD development.

One strength of this study is its multicenter study design. Our study had certain limitations. First, the study was an exploratory study with a relatively small sample size. Second, the cross-sectional study design made it impossible to evaluate whether there was a causal relationship between FLP-CGM-derived metrics and arterial stiffness. We only used arterial stiffness as a marker for the risk of developing CVD. In this regard, we are currently conducting a long-term follow-up study in the same cohort that focuses on FLP-CGM-derived metrics and onset of primary CVD or changes in arterial stiffness. Third, FLP-CGM-derived metrics were evaluated based on FLP-CGM measurements during a limited time. Due to this limitation, FLP-CGM-derived metrics related to inter-day glucose variability may be not associated with arterial stiffness after adjusting for various atherosclerotic risk factors. Repeated FLP-CGM measurements may be required to clarify the relationship between inter-day glucose variability and arterial stiffness. Fourth, we only recruited Japanese patients with type 2 diabetes. These constraints may limit the generalizability of our results. Fifth, the effects of potential confounders should be interpreted with caution. It is worthwhile to assess associations between CGM-derived metrics and baPWV stratified by gender to eliminate the potential confounding effect of gender-related factors on arterial stiffness [40]. However, this cross-sectional study included a relatively small sample with insufficient power to assess associations stratified by gender. There were no differences in baPWV between males and females (p = 0.508) (Table 1). On the other hand, the percentage of smokers was lower in the high arterial stiffness group than in the low arterial stiffness group, consistent with a previous study [13]. Although smoking is a risk factor for CVD, previous data regarding the association between smoking and arterial stiffness are inconclusive [12, 13, 26]. In a multivariate analysis that included potential conventional risk factors, neither gender nor smoking status was significantly associated with either baPWV or high arterial stiffness (data not shown), consistent with previous studies [12, 26]. Accordingly, gender and smoking status were unlikely to have had a major impact on our main findings. Furthermore, we made efforts to control for such confounding factors in multivariate regression analysis. On the other hand, some potential conventional risk factors for arterial stiffness such as fasting hyperglycemia or insulin resistance [31, 41, 42] were not included in the multivariate regression analysis. Finally, multiple anti-diabetic agents including sodium-glucose cotransporter 2 inhibitors [43–46], but not all anti-diabetic agents [47], have been shown to improve arterial stiffness. In this regard, FLP-CGM-derived metrics related to intra-day glucose variability such as SD and MAGE, and metrics related to hyperglycemia such as TAR > 13.9 mmol/L and HBGI, were still significantly associated with high arterial stiffness, even after adjusting for the use of anti-diabetic agents (Additional file 1: Table S2). However, we could not completely rule out the possible effects of anti-diabetic agents because of the cross-sectional nature of the present study. Future studies are needed to clarify these points.

## Conclusion

In this study, we demonstrated that FLP-CGM-derived metrics related to intra-day glucose variability, hyperglycemia, and hypoglycemia are significantly associated with arterial stiffness, even after adjusting for various risk factors in patients with type 2 diabetes with no history of apparent CVD (Additional file 2: Figure S1).

In addition, based on FLP-CGM-derived metrics, high postprandial glucose excursion amplitude can identify subjects who are at high risk for developing CVD. Thus, these metrics could provide medical professionals with useful information for assessing the risk of CVD.

## Supplementary information

**Additional file 1: Table S1.** List of sites and investigators. **Table S2.** Associations between FLP-CGM–derived metrics and high arterial stiffness after further adjusting for the use of anti-diabetic agents.

**Additional file 2: Figure S1.** A visual summary.

## Data Availability

The analyzed datasets are available from the corresponding author on reasonable request.

## Data availability

All data generated or analyzed during this study are included in this published article or in the data repositories listed in the References.
