# Supplementary material for: Associations between continuous glucose monitoring-derived metrics and arterial stiffness in Japanese patients with type 2 diabetes
Source: Cardiovasc Diabetol. 2021 Jan 7;20:15. doi: 10.1186/s12933-020-01194-2 (PMC7792328; doi:10.1186/s12933-020-01194-2)
Supplement: Supplementary file 1 — Additional file 1: Table S1. List of sites and investigators. Table S2. Associations between FLP-CGM–derived metrics and high arterial stiffness after further adjusting for the use of anti-diabetic agents. [file 12933_2020_1194_MOESM1_ESM.doc]

Supplementary Table 1. List of sites and investigators

| Site | Investigator(s) |
| --- | --- |
| Ashiya Central Hospital | Koji Matsushita, Manabu Narisawa |
| Aso Clinic | Katsumi Aso, Yuko Ando, Fumihiko Sato |
| Hagiwara Central Hospital | Emiko Morita, Keiichi Torimoto |
| Hayashi Clinic | Isao Hayashi |
| Inokuchi Clinic | Nobuo Inokuchi |
| Japan Community Health Care Organization Osaka Hospital | Masahiro Hatazaki, Arichika Deguchi, Azusa Shiraki |
| Juntendo Tokyo Koto Geriatric Medical Center (Department of Medicine, Diabetology and Endocrinology) | Ayako Kitamura, Eri Tanabe, Hidenori Yoshii, Tomio Onuma, Tomo Nakajima |
| Juntendo University Graduate School of Medicine (Department of Metabolism & Endocrinology) | Eisuke Yasunari, Hideyoshi Kaga, Hiroaki Sato, Hirotaka Watada, Kagemi Takeno, Luka Suzuki, Miwa Himuro, Syuhei Aoyama, Takashi Funayama, Takehiro Katahira, Takeshi Miyatsuka, Tomoya Mita, Yuya Nishida |
| Juntendo University Nerima Hospital (Department of Medicine, Diabetes and Endocrinology) | Koji Komiya |
| Kanda Naika Clinic | Satoshi Kawashima |
| Kansai Rosai Hospital (Diabetes and Endocrinology) | Tsunehiko Yamamoto |
| Kihara Diabetes Clinic | Yasuyuki Kihara |
| Kosugi Medical Clinic | Keisuke Kosugi |
| Kawasaki Hospital (Department of Internal Medicine) | Akihito Otsuka, Jun Murai |
| Matsuoka Medical Clinic | Hirofumi Matsuoka |
| Misaki Naika Clinic | Nobuichi Kuribayashi |
| Japan Labour Health and Safety Organization Kyushu Rosai Hospital, Moji Medical Center (Department of Internal Medicine) | Tadashi Arao, Kei Sugai |
| Musashino Family Clinic | Yuichi Kojima |
| Nakakinen Clinic | Maiko Nakata, Miyoko Saito, Takeshi Osonoi, Yusuke Osonoi |
| Nakama Municipal Hospital | Kohei Uriu, Yoshifumi Inada, Kanako Suzuka, Ichiro Takagi |
| National Hospital Organization Osaka National Hospital (Diabetes Center) | Ken Kato |
| Nishida Keiko Diabetes Clinic | Keiko Nishida, Akira Kurozumi, Fumi Uemura, Keiichi Torimoto, Maiko Hajime, Manabu Narisawa, Satomi Sonoda, Kumiko Tidiwa |
| Osaka General Medical Center (Department of Diabetes and Endocrinology) | Yohei Fujita, Sayoko Shimizu, Masahisa Hata, Yutaka Umayahara |
| Osaka Police Hospital (Department of Endocrinology and Metabolism) | Tetsuyuki Yasuda |
| Osaka Rosai Hospital | Kayoko Ryomoto |
| Osaka University Graduate School of Medicine (Department of Metabolic Medicine) | Iichiro Shimomura, Naoto Katakami, Takaaki Matsuoka, Mitsuyoshi Takahara, Kazuyuki Miyashita, Hiroyo Ninomiya, Naohiro Taya |
| Sasaki Hospital | Shinichiro Mine, Kenji Koikawa |
| School of Medicine, University of Occupational and Environmental Health, Japan (First Department of Internal Medicine) | Yosuke Okada, Akira Kurozumi, Manabu Narisawa, Maiko Hajime, Fumi Uemura, Satomi Sonoda, Kenichi Tanaka, Takashi Otsuka, Kenji Koikawa, Megumi Miyazaki, Akemi Tokutsu, Momoko Habu, Momo Saito, |
| Secomedic Hospital | Satomi Wakasugi, Tomoya Mita |
| Shiraiwa Medical Clinic | Toshihiko Shiraiwa |
| Taneda Clinic | Yoshinobu Taneda |
| Takaishi Naika Ichoka Clinic | Tomoya Mita |
| Tobata General Hospital (Department of Internal Medicine) | Kazuko Kanda |
| Wakamatsu Hospital of the University of Occupational and Environmental Health | Keiichi Torimoto, Kosuke Nishio |

Sites and investigators are listed in alphabetical order.

**Supplementary Table 2.** Associations between FLP-CGM–derived metrics and high arterial stiffness after further adjusting for the use of anti-diabetic agents.

| Parameter | Odds ratio (95% CI) | p value |
| --- | --- | --- |
| Mean glucose (1 mmol/L increase) | 1.19 (0.95-1.50) | 0.131 |
| SD (mmol/L) (1 mmol/L increase) | 1.76 (1.07-2.89) | 0.026 |
| CV (%) (1% increase) | 1.04 (0.99-1.09) | 0.093 |
| MAGE (1 mmol/L increase) | 1.24 (1.08-1.42) | 0.003 |
| TIR^3.9–10 mmol/L^ (10% increase) | 0.87 (0.72- 1.07) | 0.197 |
| TAR^>10 mmol/L^ (1% increase) | 1.15 (0.99-1.04) | 0.161 |
| TAR^>13.9 mmol/L^ (1% increase) | 1.04 (1.01-1.08) | 0.025 |
| TBR^<3.9 mmol/L^ (1% increase) | 0.99 (0.93-1.06) | 0.783 |
| TBR^<3.0 mmol/L^ (1% increase) | 0.95 (0.78-1.16) | 0.615 |
| LBGI (1 unit increase) | 0.95 (0.79-1.13) | 0.533 |
| HBGI (1 unit increase) | 1.09 (1.01-1.18) | 0.032 |
| MODD (1 mmol/L increase) | 1.29 (0.74-2.23) | 0.373 |
| IQR (1mmol/L increase) | 1.30 (0.83-2.04) | 0.252 |
| HbA1c (1% increase) (excluding HbA1c) | 0.90 (0.61-1.32) | 0.581 |

Adjusted for age, gender, body mass index, duration of diabetes, HbA1c, systolic blood pressure, total cholesterol, high-density lipoprotein cholesterol, logarithm of triglycerides, serum uric acid, estimated glomerular filtration rate, logarithm of urinary albumin excretion, presence of diabetic retinopathy, smoking status (never smoker, previous smoker, or current smoker), use of insulin therapy, use of sodium-glucose cotransporter 2 inhibitors, use of biguanides, use of dipeptidyl peptidase 4 inhibitors, use of thiazolidinediones, use of sulfonylureas, use of alpha-glucosidase inhibitors, use of glinides, use of angiotensin-converting enzyme inhibitors and/or angiotensin II receptor blockers, use of statins, and use of anti-platelet agents

CI, confidence interval; CV, coefficient of variation; FLP-CGM, FreeStyle Libre Pro continuous glucose monitoring; HBGI, high blood glucose index; IQR, interquartile range; LBGI, low blood glucose index; MAGE, mean amplitude of glycemic excursion; MODD, mean of daily differences; SD, standard deviation; TAR, time above range; TBR, time below range; TIR, time in range.

**Supplementary Figure Legend**

**Figure 1.**

FLP-CGM–derived metrics related to intra-day glucose variability, hyperglycemia, and hypoglycemia are significantly associated with arterial stiffness in patients with type 2 diabetes.

CV, coefficient of variation; CGM, continuous glucose monitoring; HBGI, high blood glucose index; MAGE, mean amplitude of glycemic excursion; SD, standard deviation; TAR, time above range; TBR, time below range; TIR, time in range.
