# Supplementary material for: Associations between continuous glucose monitoring-derived metrics and arterial stiffness in Japanese patients with type 2 diabetes
Source: Cardiovasc Diabetol. 2021 Jan 7;20:15. doi: 10.1186/s12933-020-01194-2 (PMC7792328; doi:10.1186/s12933-020-01194-2)
Supplement: Supplementary file 2 — Additional file 2: Figure S1. A visual summary. [file 12933_2020_1194_MOESM2_ESM.pptx]

## Slide 1
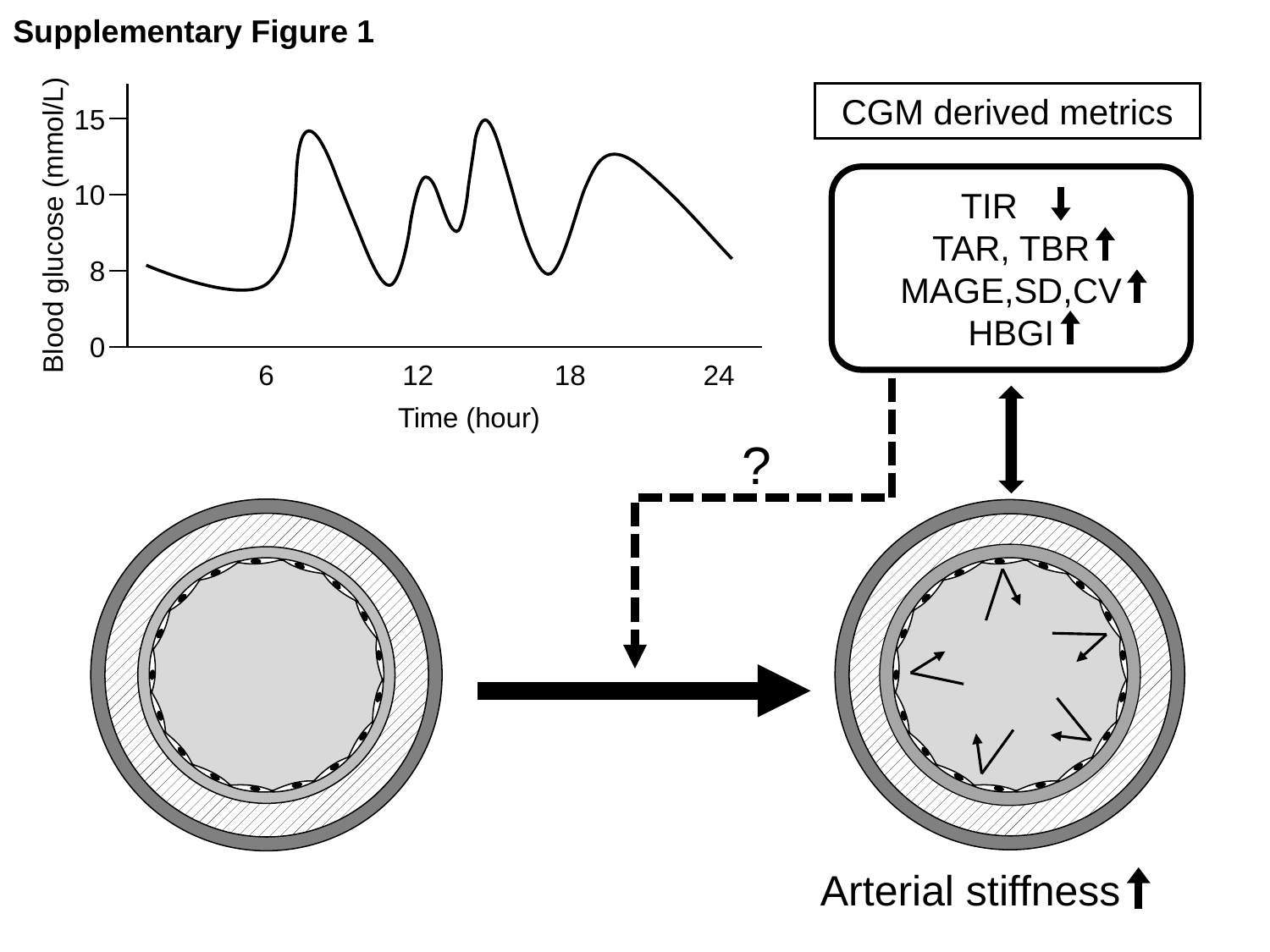

Supplementary Figure 1
CGM derived metrics
15
TIR
TAR, TBR
MAGE,SD,CV
HBGI
10
Blood glucose (mmol/L)
8
0
6
12
18
24
Time (hour)
?
Arterial stiffness
